# Closed-circuit bioelectrochemical operation is associated with a higher relative contribution of deterministic assembly and altered targeted functional-gene profiles in soil microbiomes

**DOI:** 10.3389/fmicb.2026.1815770

**Published:** 2026-10-09

**Authors:** Pengyu Zhao, Shuhui Zhang, Xiaodong Zhao

**Affiliations:** 1 College of Biological Sciences and Technology, Taiyuan Normal University, Jinzhong, China; 2 Soil Health Laboratory in Shanxi Province, Taiyuan, China; 3 Shanxi Key Laboratory of Earth Surface Processes and Resource Ecology Security in Fenhe River Basin, Taiyuan Normal University, Jinzhong, China; 4 Institute of Eco-environment and Industrial Technology, Shanxi Agricultural University, Taiyuan, China

**Keywords:** closed-circuit operation, microbial association networks, microbial fuel cells, soil microbial community assembly, targeted functional genes

## Abstract

Microbial fuel cells (MFCs) are increasingly explored for soil remediation, yet the microbial ecological responses associated with conductive-material interfaces and closed-circuit operation remain incompletely understood. Using three experimental setups—a non-electrode anaerobic control (AT), an open-circuit conductive-material treatment (TO), and a closed-circuit treatment (TC)—we examined bacterial community composition, assembly processes, targeted functional-gene profiles, and exploratory co-occurrence patterns. The TO treatment showed a more highly connected exploratory network and slightly lower diversity within the targeted functional-gene panel than AT. The TC treatment showed a higher estimated relative contribution of deterministic processes than TO, although stochastic processes remained dominant in all treatments. TC was also characterized by several electroactive-associated taxa, including *Geobacteraceae* and *Anaeromyxobacter*, and by higher 16S-normalized relative abundances of several sulfur-cycling genes. These results suggest that the introduction of conductive materials and closed-circuit operation were associated with different bacterial community and targeted functional-gene responses. Because the TO–AT contrast includes both electrodes and carbon fibers, reactor-level replication was limited, and electrochemical output was not continuously quantified, the findings should be interpreted as exploratory associations rather than direct evidence of current-driven metabolic specialization. This study provides testable hypotheses for future bioelectrochemical soil-remediation experiments with greater reactor replication, electrochemical monitoring, and process-rate measurements.

## 1 Introduction

Soil contamination by persistent anthropogenic pollutants, including antibiotics and agrochemicals, poses a widespread threat to agricultural sustainability by disrupting biogeochemical cycling and microbial functions. While conventional physicochemical remediation strategies—such as excavation and chemical oxidation—can effectively abate pollutant loads, their applicability is often constrained by prohibitive costs, high energy demands, and the risk of secondary ecological disruption (Aparicio et al., 2022; Grifoni et al., 2022). Consequently, soil remediation strategies are increasingly shifting toward low-energy, *in situ* biological approaches.

Previous studies have demonstrated that soil bioelectrochemical systems can alter microbial community composition under open- and closed-circuit conditions and can influence the removal of antibiotics and antibiotic resistance genes (Zhao et al., 2019; Song et al., 2022; Zhang et al., 2020). However, differences in taxonomic composition alone do not resolve how community assembly processes or targeted biogeochemical functional-gene profiles vary among these experimental configurations. Previous studies of soil bioelectrochemical systems have primarily focused on microbial composition, electroactive taxa, contaminant removal, and associated biogeochemical transformations (Zhao et al., 2019; Zhang et al., 2020; Song et al., 2022), whereas null-model-based partitioning of stochastic and deterministic community assembly has been less frequently evaluated in this experimental context. Stochastic and deterministic processes provide a complementary ecological framework for interpreting microbial community organization beyond taxonomic composition alone (Stegen et al., 2013; Yuan et al., 2019; Zhou and Ning, 2017). In parallel, gene-based profiles associated with C, N, P, and S cycling can provide complementary information on microbial functional potential (Zhu et al., 2024; Chen et al., 2025), although DNA-level gene abundance should not be interpreted as direct evidence of gene expression or biogeochemical process rates (Louca et al., 2018).

Accordingly, rather than asking whether open- and closed-circuit systems differ in microbial composition *per se*, the present study examined whether these experimental configurations were associated with differences in bacterial community assembly and targeted functional-gene profiles under tetracycline stress. We established a non-electrode anaerobic control (AT), an open-circuit treatment containing electrodes and short-cut carbon fibers (TO), and a closed-circuit treatment with the same conductive-material configuration as TO (TC). The AT–TO contrast represents the combined association with conductive-material addition, whereas the TO–TC contrast evaluates responses associated with circuit closure under otherwise identical conductive-material configurations. We assessed bacterial taxonomic composition, null-model-based community assembly following established ecological frameworks (Stegen et al., 2013; Yuan et al., 2019), characteristic taxa, exploratory association-network topology, and the relative abundances of 71 targeted functional genes involved in C, N, P, and S cycling.

## 2 Materials and methods

### 2.1 Soil sampling

The experimental soil, classified as yellow loam, was collected from the tillage layer (0–20 cm depth) of an agricultural field in Taigu District, Jinzhong City, Shanxi Province, China (37°46′N, 112°58′E). Following collection, soil samples were air-dried at room temperature in a shaded, well-ventilated laboratory environment and then sieved through a 2-mm mesh to remove debris and homogenize the soil. The processed soil was thoroughly mixed to ensure homogeneity prior to experimental use.

The baseline physicochemical properties of the prepared soil were as follows: pH 8.55, electrical conductivity (EC) 126.07 μS cm?^1^, available phosphorus (AP) 4.09 mg kg?^1^, and alkali-hydrolyzable nitrogen (AN) 64.17 mg kg?^1^. Soil physicochemical parameters were determined using standard analytical methods as described in the literature. It should be noted that the EC value reported here represents the salinity background of the soil matrix measured under standard air-dried conditions and does not reflect the *in situ* pore water conductivity during reactor operation, which was not measured in this study.

### 2.2 Soil MFC construction and operation

Three experimental configurations were established: a closed-circuit treatment (TC), an open-circuit conductive-material treatment (TO), and a non-electrode anaerobic control (AT) (Figure 1). The TC reactors were assembled following the design of Zhang et al. (2020), incorporating a vertically aligned activated carbon air-cathode paired with a carbon cloth anode. The carbon cloth anode (L 56.5 cm × W 5 cm) was pretreated by soaking in acetone overnight, followed by sequential rinsing with ethanol and distilled water (three times each), and then placed on the periphery of the reactor (Li et al., 2016). The activated carbon air-cathode (L 10 cm × W 5 cm) was fabricated in the laboratory using a rolling-press method without commercial suppliers, as described previously (Zhang et al., 2014). Briefly, the cathode consisted of a catalyst layer (activated carbon), a gas diffusion layer (carbon black), and a stainless steel mesh current collector. The finished cathode was wrapped around a plastic tube (L 6 cm × D 3 cm) with equidistant small holes (D ≈ 2 mm), then inserted into the center of the reactor. An external resistor of 100 Ω was connected between the anode and cathode of the TC reactors to establish the closed-circuit configuration.

**Figure 1:**
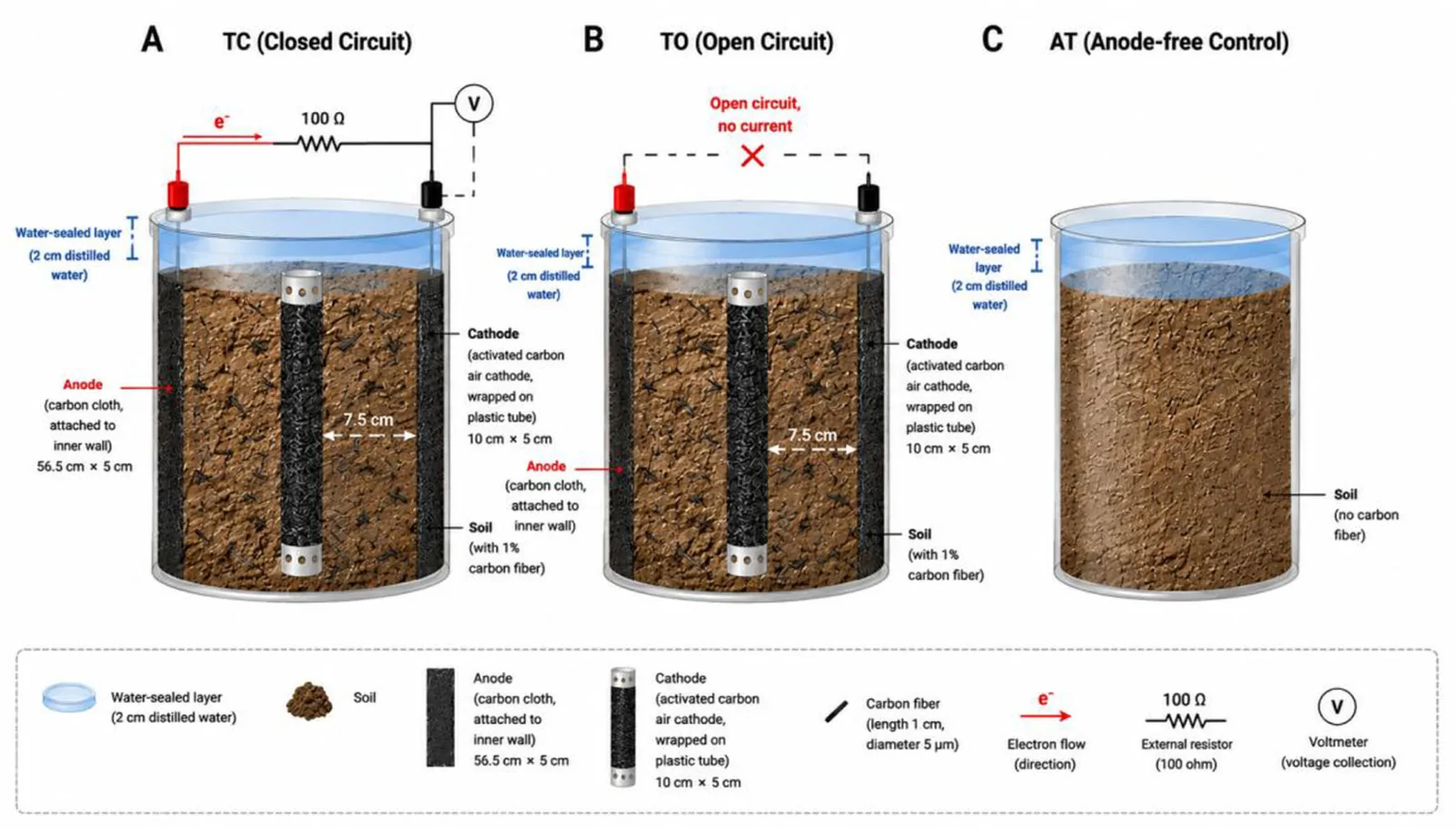
Schematic diagram of the experimental soil microbial fuel cell (soil-MFC) systems under different treatments. **(A)** TC, closed-circuit treatment: the cylindrical reactor consisted of an outer anode made of carbon cloth attached to the inner wall of the reactor and a central cathode composed of an activated carbon air-cathode wrapped around a plastic tube. The electrode spacing was 7.5 cm. Soil was mixed with 1% carbon fiber and covered with a 2 cm distilled water-sealed layer. The electrodes were connected through an external resistor (100 Ω) to establish the closed-circuit configuration. **(B)** TO, open-circuit conductive-material treatment: identical physical configuration to TC, including electrodes and carbon fiber addition, but operated under open-circuit conditions with no external-circuit connection. **(C)** AT, non-electrode anaerobic control: reactor containing only soil and a 2 cm water-sealed layer, without electrodes or carbon fiber addition.

Each reactor was loaded with 1,500 g of soil and 600 mL of deionized water, supplemented with tetracycline at 20 mg/kg (purity: HPLC, Shanghai Macklin Biochemical Technology Co., Ltd., China) to simulate tetracycline stress-induced microbial community assembly (Zhai et al., 2026; Zhao et al., 2024). No external carbon sources or nutrients were added throughout the experiment. The sole carbon and energy sources for microorganisms were the indigenous organic matter present in the soil. Specifically, tetracycline concentrations in agricultural soils near feedlots have been reported to range from 5 to 20 mg/kg (Ji et al., 2012; Shu et al., 2025), and can reach up to 112 mg/kg in soils irrigated with sewage (Zhao et al., 2024). Therefore, the dosage of 20 mg/kg used in this study falls within the range of environmentally relevant concentrations, representing a scenario of moderate to severe tetracycline contamination. This study aims to investigate microbial community assembly processes under tetracycline stress.

To enhance soil conductivity, short-cut carbon fibers (length 1 cm, diameter 5 μm) were pretreated with acetone following the same procedure as for the carbon cloth and then thoroughly mixed into the soils of the TC and TO treatments (but not the AT control) at a mass fraction of 1% (Li et al., 2016). The AT control received no electrodes or carbon fibers. These carbon fibers served solely as conductive materials to reduce the internal resistance of the soil and were not intended as an additional carbon source for microbial metabolism. In contrast to AT, both TC and TO were equipped with identical electrode materials (a carbon cloth anode and an activated carbon air-cathode) and the same amount of carbon fibers. The sole operational distinction between TC and TO was circuit status: TC was connected through a 100 Ω external resistor, whereas TO was maintained under open-circuit conditions. Therefore, the TC–TO comparison represents responses associated with closed-circuit operation rather than responses to a quantified electrical dose. The electrodes in TO provided conductive surfaces for microbial colonization, but the external circuit remained open. Thus, TO represents the conductive-material configuration under open-circuit conditions, whereas TC represents the same conductive-material configuration under closed-circuit conditions.

After soil packing, a 2-cm layer of distilled water was maintained above the soil to sustain anaerobic conditions, with daily replenishment of evaporated water using a syringe. The AT was designated as the anaerobic control, consistent with previous studies (Zhai et al., 2026). All reactors were incubated in the dark at 30 °C for 92 days, mimicking the typical temperature and light conditions of subsurface soils. Microbial community, functional-gene, and physicochemical analyses were based exclusively on samples collected at the end of the 92-day incubation. No intermediate time points were sampled; therefore, the present study evaluates endpoint differences among experimental configurations rather than temporal microbial succession. Given the cylindrical configuration of the reactor, with the activated carbon air-cathode positioned at the center and the carbon cloth anode wrapped around the periphery, the soil between them formed an annular zone. Soil samples were collected along the radial direction from the cathode to the anode, specifically from the cathode surface (C), the middle point (M), and the anode surface (A), as illustrated in Figure 1. All samples were collected from a depth of 2–4 cm below the soil surface to avoid the disturbance of surface-dissolved oxygen, following the sampling strategy described by Zhang et al. (2020). Each soil sample was divided into two portions: one was air-dried for physicochemical measurements, and the other was immediately frozen at −80 °C for subsequent high-throughput sequencing (16S rRNA gene amplicon) and high-throughput quantitative PCR (HT-qPCR) analysis of functional genes.

For downstream analyses, AT comprised three independently prepared soil units. TO and TC each comprised two independently operated reactors, from which three spatial subsamples were collected at the cathode-facing region, midpoint, and anode-facing region. The reactor, rather than the spatial subsample, was considered the experimental unit for treatment-level inference. The spatial subsamples were retained to characterize within-reactor heterogeneity and were not treated as independent biological replicates.

### 2.3 Soil physicochemical properties measurements

Air-dried soil samples were utilized for the assessment of physicochemical properties. Soil pH and EC were measured potentiometrically in a soil-to-water suspension at a 1:2.5 (w/v) ratio. AP was quantified using the molybdenum-antimony anti-colorimetric method, while AN was determined via the alkaline hydrolysis diffusion method (Bao, 2000).

### 2.4 DNA extraction, PCR amplification, and high-throughput sequencing

Total genomic DNA was extracted from 1.0 g of fresh soil using the E.Z.N.A.^®^ Soil DNA Kit (Omega Bio-tek, Norcross, GA, USA) according to the manufacturer's protocol. DNA quality was evaluated by 1% agarose gel electrophoresis, and the purity and concentration were determined using a microplate spectrophotometer. Samples with an absorbance ratio (A260/A280) between 1.8 and 2.0 were retained for downstream analyses. The V4 hypervariable region of the bacterial 16S rRNA gene was amplified using primers 515F (5′-GTGCCAGCMGCCGCGGTAA-3′) and 806R (5′-GGACTACHVGGGTWTCTAAT-3′). PCR reactions were carried out in 20 μL mixtures containing 4 μL of 5 × FastPfu Buffer, 2 μL of dNTPs (2.5 mM), 0.8 μL of each primer (5 μM), 0.4 μL of DNA polymerase, 0.2 μL of BSA, 10 ng of template DNA, and ddH_2_O to the final volume. Thermal cycling was performed as follows: initial denaturation at 98 °C for 1 min; 30 cycles of denaturation at 98 °C for 10 s, annealing at 50 °C for 30 s, and extension at 72 °C for 30 s; followed by a final extension at 72 °C for 5 min. Amplified products were pooled and sent to Majorbio Bio-Pharm Technology Co., Ltd. (Shanghai, China) for high-throughput sequencing.

Raw paired-end reads were subjected to quality filtering to remove low-quality reads and reads containing ambiguous bases. The filtered paired-end reads were subsequently merged according to their overlapping regions, and sequences failing to meet the quality criteria were discarded. Chimeric sequences were identified and removed before downstream analysis. The resulting high-quality, non-chimeric sequences were clustered into operational taxonomic units (OTUs) at a 97% sequence-similarity threshold using UPARSE (v11). Representative sequences from each OTU were taxonomically classified using the RDP Classifier against the corresponding bacterial 16S rRNA gene reference database. A total of 5,763 OTUs were retained across all samples for subsequent community analyses. Good's coverage values ranged from 98.2% to 99.1%, and the rarefaction curves approached saturation, indicating adequate sequencing coverage for the present dataset.

### 2.5 Quantification of functional genes by high-throughput quantitative PCR (HT-qPCR)

HT-qPCR was employed to quantify the absolute copy numbers of the bacterial 16S rRNA gene and 71 functional genes associated with carbon (C), nitrogen (N), phosphorus (P), and sulfur (S) cycling. The 16S rRNA gene served as the internal reference for normalization.

The qPCR assays were performed in a 20-μL reaction mixture consisting of 10 μL of SYBR Green Mix, 0.5 μM of each forward and reverse primer, 2 μL of template DNA, and 7 μL of ddH_2_O. Each qPCR reaction was performed in three technical replicates (n = 3 per biological replicate). Thermal cycling was conducted on a Roche LightCycler 480 instrument (Roche Diagnostics, Basel, Switzerland) under the following conditions: initial denaturation at 95 °C for 3 min, followed by 40 cycles of denaturation at 95 °C for 15 s, annealing at 60 °C for 30 s, and extension at 72 °C for 30 s.

The gene copy numbers were calculated based on the threshold cycle C_T values using Equation 1, as previously described (Zhu et al., 2018).


$$\begin{array}{l} \text{Gene copy number}=10^{\left(31-C_{T}\right)/\left(10/3\right)} \end{array}$$

The relative abundance of each functional gene was expressed as the ratio of its copy number to that of the 16S rRNA gene (Chen et al., 2025).

The 71 functional genes have been extensively validated in previous studies (Chen et al., 2025; Mao et al., 2025), encompassing a wide range of biogeochemical processes, including carbon degradation, carbon fixation, methane metabolism, and cycling of C, N, P, and S. Detailed information on the primers used for these genes is provided in Supplementary Table S1.

### 2.6 Data analysis

The reactor was considered the independent experimental unit for treatment-level statistical inference. AT comprised three independently prepared soil units, whereas TO and TC each comprised two independently operated reactors. The three spatial subsamples collected within each TO and TC reactor represented within-reactor observations and were not treated as independent biological replicates. For treatment-level analyses of univariate variables, measurements from spatial subsamples within each reactor were first averaged to obtain one reactor-level value. Thus, treatment-level summaries were based on three independent units for AT and two independent reactors each for TO and TC. Given the limited reactor-level replication, statistical comparisons were considered exploratory, and emphasis was placed on the direction and magnitude of treatment-associated differences rather than strong causal inference.

#### 2.6.1 Exploratory bacterial association-network analysis

Exploratory bacterial association networks were constructed separately for the AT, TO, and TC configurations using the 50 most abundant OTUs. Pairwise associations among OTUs were evaluated using Spearman's rank correlation analysis (Friedman and Alm, 2012). Associations with an absolute Spearman correlation coefficient |ρ| ≥ 0.60 and *P* < 0.05 were retained for network visualization. Nodes represent bacterial OTUs, whereas edges represent retained positive or negative statistical associations between OTUs. Network topological descriptors, including the number of nodes and edges, average degree, network diameter, modularity, average path length, average weighted degree, and average clustering coefficient, were calculated, and the networks were visualized using Gephi (Gephi Consortium, Paris, France) (Bastian et al., 2009). Because reactor-level replication was limited and the treatment-specific networks included within-reactor spatial observations, this analysis was used only as an exploratory visualization of bacterial association patterns. Network topology was therefore interpreted descriptively and was not used as direct evidence of microbial interaction strength, ecological stability, or treatment-induced network restructuring.

#### 2.6.2 Calculation of standardized targeted functional-gene summary scores

To summarize coordinated variation within the targeted functional-gene panel, the 16S-normalized relative abundance of each gene was standardized across samples using Z-score transformation. Mean standardized scores were calculated across all detected target genes and separately for genes assigned to C-, N-, P-, and S-cycling categories. These scores were used solely as descriptive summaries of the measured gene panel and do not represent gene expression, biogeochemical process rates, or the total functional capacity of the microbial community. Equal weighting of genes represents an analytical simplification and was considered when interpreting the results (Jing et al., 2015; Wagg et al., 2014).

## 3 Results

### 3.1 Variations in soil physicochemical properties

Soil physicochemical properties exhibited distinct responses to the different treatments. Soil pH and EC remained stable, with no significant differences detected among treatments (*P* > 0.05). In contrast, the concentrations of available nutrients were significantly affected (*P* < 0.05). Specifically, relative to the AT, AP was markedly reduced in both the TO and TC treatments, with decreases of 48.13% and 45.77%, respectively. Conversely, the AN content in the TO treatment increased substantially by 86.98%. These results indicate treatment-associated differences in endpoint AP and AN concentrations, whereas pH and EC did not differ significantly among treatments (Figure 2).

**Figure 2:**
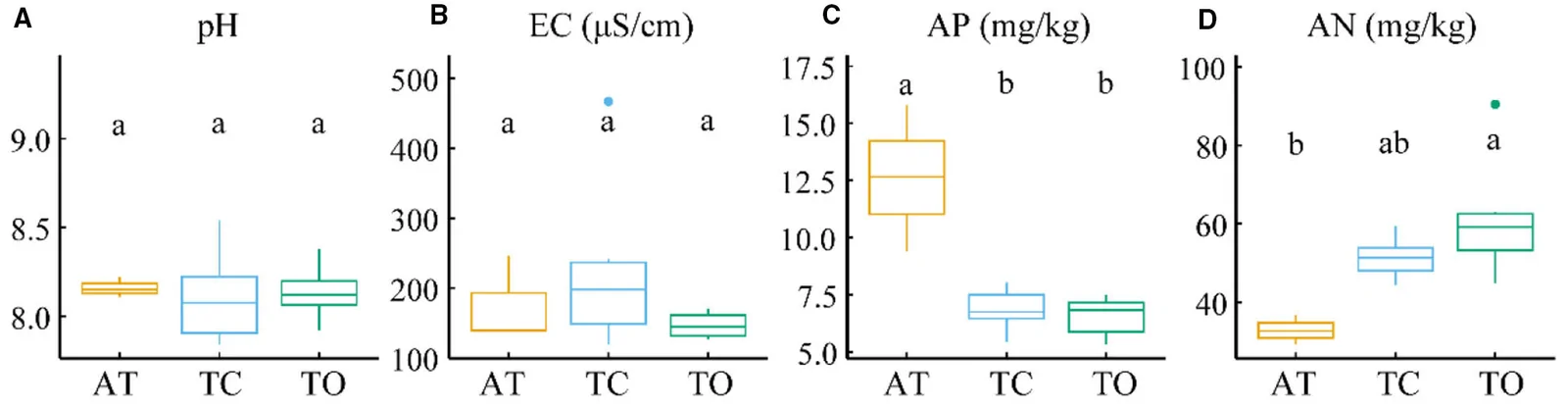
Variations in soil physicochemical properties across different treatments in the microbial fuel cell system. **(A)** Soil pH. **(B)** Soil electrical conductivity (EC). **(C)** Soil available phosphorus (AP) content. **(D)** Soil alkali-hydrolyzable nitrogen (AN) content. AT, non-electrode anaerobic control; TO, open-circuit conductive-material treatment; TC, closed-circuit treatment. Different lowercase letters indicate statistically significant differences among treatments (*P* < 0.05).

### 3.2 Composition and assembly mechanisms of soil bacterial communities

High-throughput sequencing revealed treatment-associated differences in bacterial taxonomic composition (Figure 3A; Supplementary Figure S1). Supplementary Figure S1 provides complementary comparisons of bacterial taxa at the phylum and genus levels, whereas the community-assembly analysis and β-diversity decomposition are presented in Figures 3B–F, respectively. As shown in Figure 3A, Proteobacteria (21%), Acidobacteriota (16.78%), and Actinobacteriota (16.63%) were the three dominant phyla across the experimental systems.

**Figure 3:**
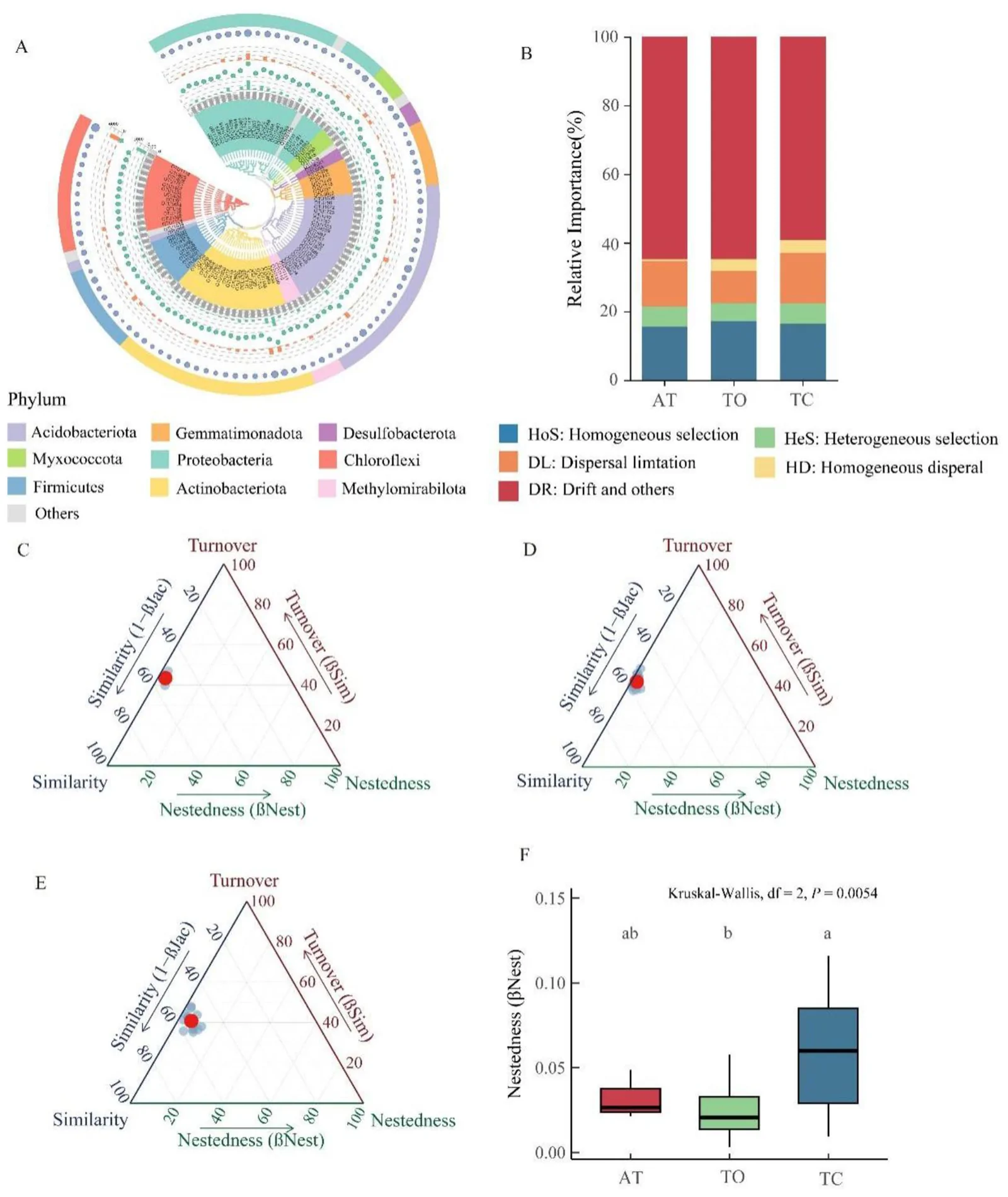
Variations in the structure and assembly mechanisms of soil bacterial communities across different treatments in the microbial fuel cell system. **(A)** Taxonomic composition of soil bacterial communities. **(B)** Assembly processes of soil bacterial communities. **(C–E)** Beta-diversity decomposition of bacterial communities in the **(C)** AT, **(D)** TO, and **(E)** TC treatments. **(F)** Variations in the nestedness component (βNest) among different treatments. Different lowercase letters indicate significant differences among treatments (*P* < 0.05).

Further analysis revealed significant variations in the relative abundances of specific taxa among the different treatments. As shown in Supplementary Figure S1A, the relative abundances of Gemmatimonadota and Bacteroidota were lower in TC than in AT, whereas WPS-2 showed a higher relative abundance in TC (P < 0.05). At the genus level (Supplementary Figure S1B), Skermanella and Archangium showed lower relative abundances in TC than in AT, whereas Thermincola showed a higher relative abundance in TO.

Beta-diversity decomposition (Figures 3C–F) highlighted significant disparities in the nestedness component (βNest) among treatments. Specifically, the βNest index was significantly higher in the TC treatment than in the TO group (*P* < 0.05). To elucidate the assembly mechanisms, null model analyses based on phylogenetic binning (βNRI and RC_bray) were conducted. Null-model analysis indicated that stochastic processes remained dominant in all treatments, although TC showed a higher estimated relative contribution of deterministic processes than TO (Figure 3B), indicating a difference in the relative balance of assembly processes under the closed-circuit configuration.

In summary, although electrode presence and closed-circuit operation did not substantially alter the overall dominance of major phyla, the higher βNest value in TC indicates a stronger nested component of compositional dissimilarity. This pattern is consistent with greater taxon loss or subset formation under closed-circuit conditions, but it does not independently identify environmental filtering. The inference of an increased deterministic contribution is based primarily on the null-model analysis.

To further identify characteristic taxa within the MFC systems, the specificity and occupancy of individual OTUs were evaluated. As shown in Figure 4, a total of 35, 24, and 19 characteristic OTUs were identified in the AT, TO, and TC treatments, respectively. Notably, the TC treatment was characterized by the presence of several known electroactive bacteria (EAB) among its characteristic taxa, including *Geobacteraceae, Anaeromyxobacter*, and *Thermincola*.

**Figure 4:**
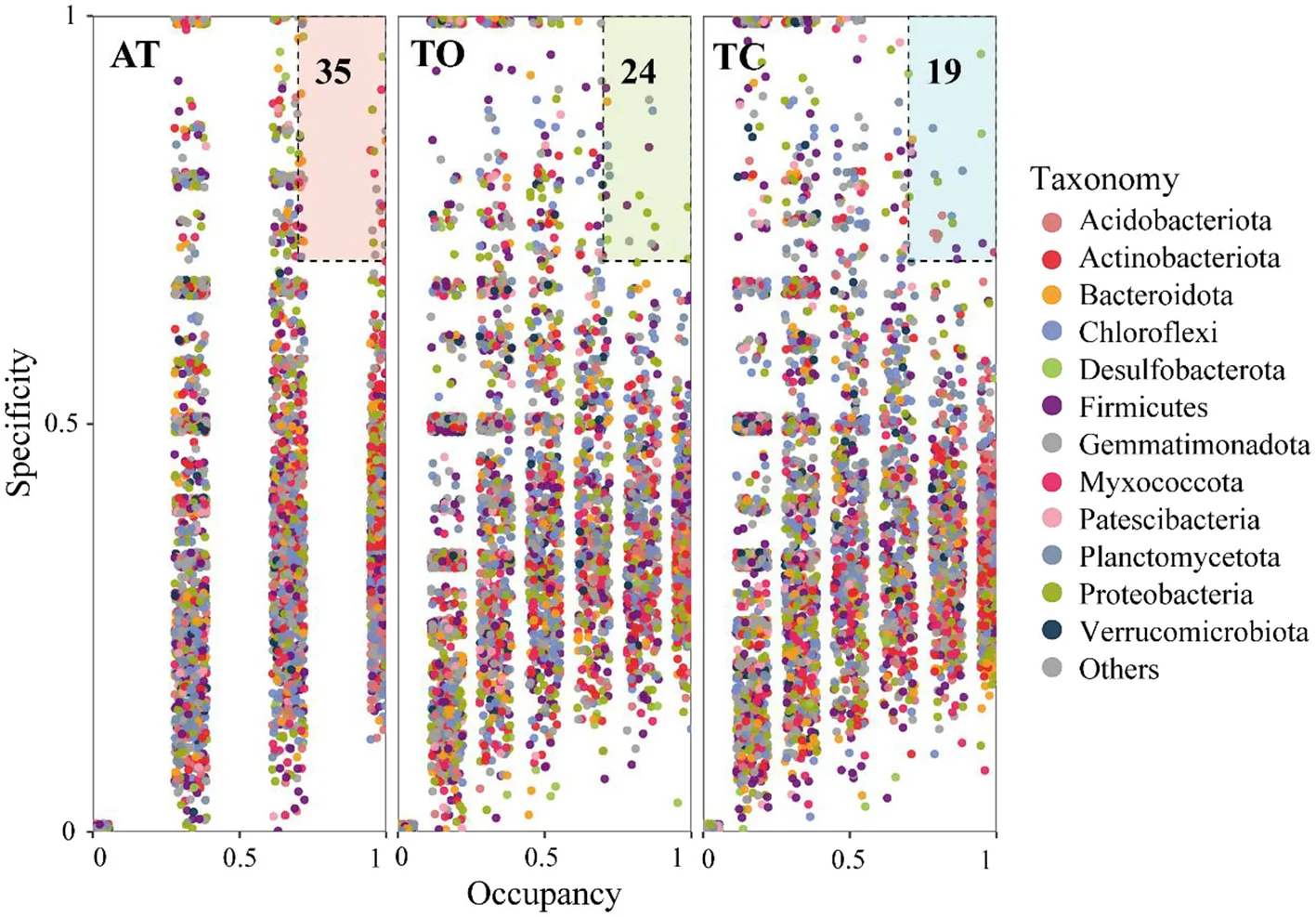
Distribution of characteristic bacterial taxa across the three experimental configurations. The specificity-occupancy plots illustrate the distribution and specificity of OTUs with a relative abundance > 0.01%. OTUs exhibiting both specificity and occupancy > 0.7 were defined as characteristic taxa.

Co-occurrence network analysis revealed descriptive differences in bacterial association-network topology among AT, TO, and TC (Figure 5; Table 1). The TO network contained the greatest number of retained nodes (50) and edges (173) and showed the highest average degree (6.92), whereas AT contained fewer nodes (37) and edges (48). TC showed intermediate values for the number of retained nodes (41), edges (71), and average degree (3.46). In contrast, modularity was highest in AT (0.595), followed by TC (0.489) and TO (0.421). Thus, TC was more modular than TO but less modular than AT. TC also showed the greatest average path length (4.211), whereas TO showed the shortest average path length (2.319). Because these network metrics can be influenced by the number of retained nodes, network density, sample size, and the underlying association-inference procedure, the observed differences are treated as descriptive patterns rather than direct evidence of treatment-induced changes in microbial interactions, stability, or robustness.

**Figure 5:**
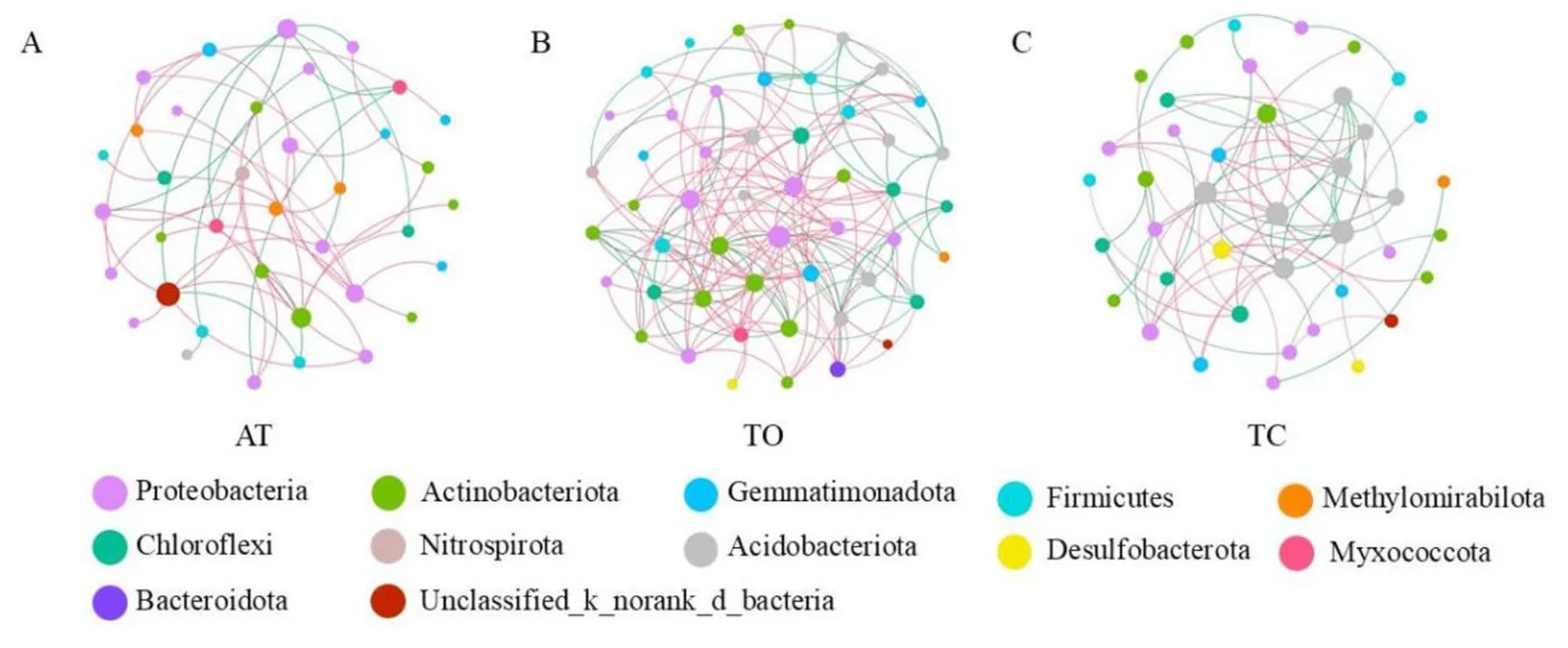
Exploratory bacterial co-occurrence networks under the three experimental configurations. Networks were constructed for **(A)** the non-electrode anaerobic control (AT), **(B)** the open-circuit conductive-material treatment (TO), and **(C)** the closed-circuit treatment (TC). Nodes represent OTUs and are colored according to phylum, whereas edges represent inferred statistical associations retained according to the specified network-construction criteria. Node size is proportional to degree. The TO network contained the greatest number of retained nodes and edges and the highest average degree. Modularity was highest in AT (0.595), intermediate in TC (0.489), and lowest in TO (0.421). These network properties are presented descriptively and should not be interpreted as direct measures of microbial interaction strength, ecological stability, or robustness.

**Table 1:** Topological properties of soil bacterial co-occurrence networks under different bio-electrochemical treatments.

Topological parameters	AT	TO	TC
Nodes	37	50	41
Edges	48	173	71
Average degree	2.595	6.92	3.463
Network diameter	9	4	10
Modularity	0.595	0.421	0.489
Average path length	3.081	2.319	4.211
Average weighted degree	2.494	6.417	3.036
Average clustering coefficient	0.119	0.386	0.324

### 3.3 Variations in functional genes across different MFC treatments

#### 3.3.1 Relative-abundance profiles of targeted functional genes

Quantitative PCR analysis revealed treatment-associated differences in the 16S-normalized relative abundances of targeted functional genes (Figure 6; Supplementary Figures S2–S5). For carbon cycling, 66.7% of the detected genes showed significantly higher relative abundances in TC than in AT and TO (Figure 6A; Supplementary Figure S2, *P* < 0.05). These genes were associated with carbon degradation, carbon fixation, and methane metabolism.

**Figure 6:**
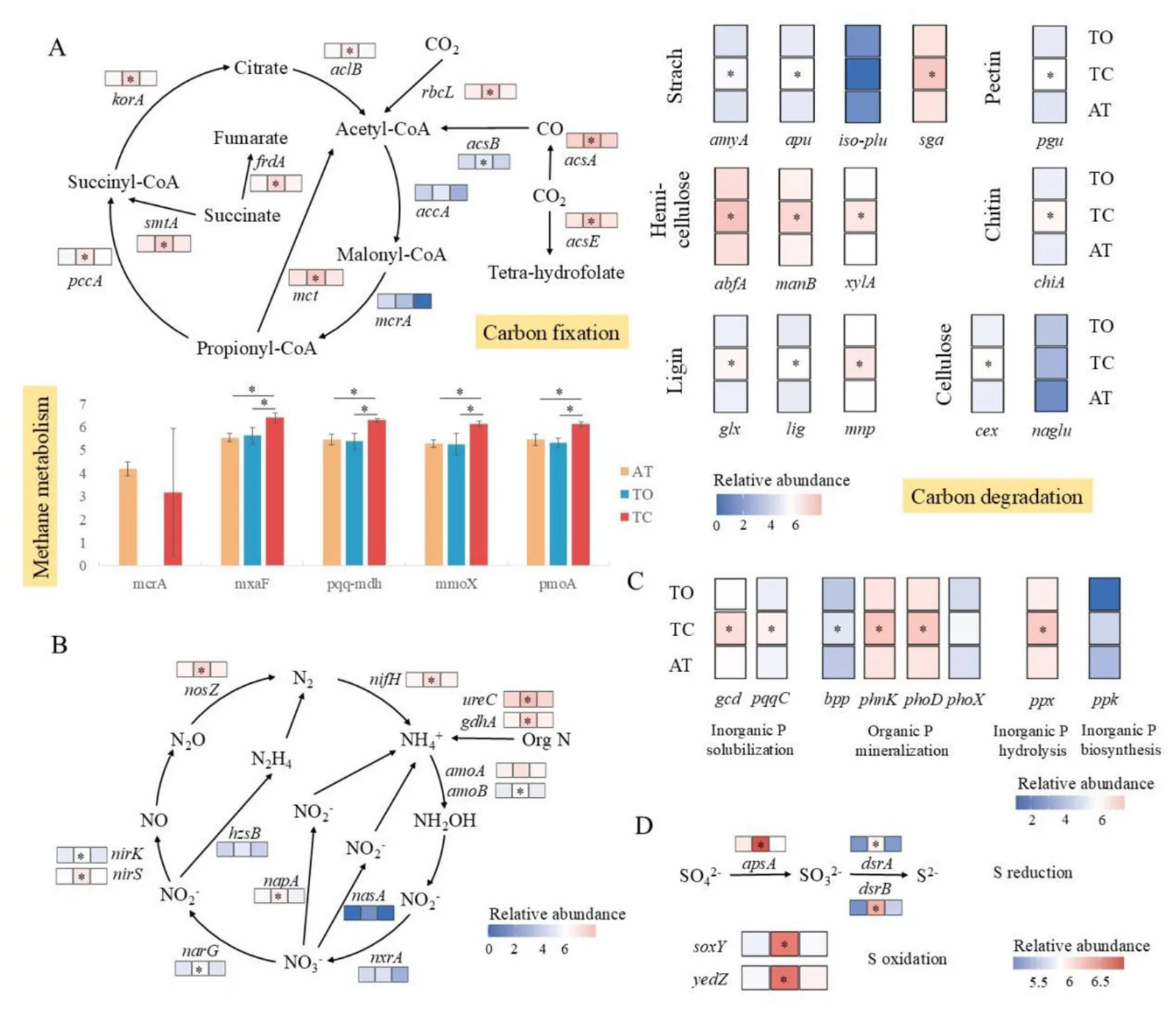
Relative abundances of targeted functional genes involved in biogeochemical cycling across the three experimental configurations. **(A)** Carbon-cycling genes associated with carbon degradation, carbon fixation, and methane metabolism. **(B)** Nitrogen-cycling genes associated with nitrification, denitrification, nitrogen fixation, ammonification, and nitrate reduction. **(C)** Phosphorus-cycling genes associated with inorganic P solubilization, organic P mineralization, P hydrolysis, and polyphosphate metabolism. **(D)** Sulfur-cycling genes associated with sulfur oxidation and sulfate reduction. Gene abundances were normalized to bacterial 16S rRNA gene copy number. Heatmap colors indicate variation in relative abundance, and bars represent the mean ± SD of the displayed sampling units. AT comprised three independently prepared soil units. TO and TC each comprised two independently operated reactors, with three spatial subsamples collected from each reactor. The reactor, rather than the spatial subsample, was considered the experimental unit for treatment-level inference. Spatial subsamples were retained to characterize within-reactor heterogeneity and were not treated as independent biological replicates. Each HT-qPCR assay was performed in three technical replicates, which were averaged before downstream analysis. Different lowercase letters indicate significant differences among treatments (*P* < 0.05). Asterisks indicate statistically significant treatment differences (*P* < 0.05).

For nitrogen cycling, 12 genes, representing 63.2% of the detected N-cycling genes, showed significantly higher relative abundances in TC than in AT and/or TO (Figure 6B; Supplementary Figure S3, *P* < 0.05). These genes included targets associated with denitrification (narG, nirS, nirK, and nosZ), nitrification (amoA and amoB), nitrogen fixation (nifH), and organic nitrogen transformation (ureC and gdhA).

For phosphorus cycling, six genes, representing 75% of the detected P-cycling genes, showed significantly higher relative abundances in TC than in the other treatments (Figure 6C; Supplementary Figure S4, *P* < 0.05). These genes were associated with inorganic P solubilization (gcd and pqqC), organic P mineralization (bpp, phoD, and phoX), and other measured P-transformation functions.

Sulfur-cycling genes showed a consistent treatment-associated pattern. All five detected S-cycling genes (apsA, dsrA, dsrB, soxY, and yedZ) had significantly higher relative abundances in TC than in AT and TO (Figure 6D; Supplementary Figure S5, *P* < 0.05). These results indicate differences in the relative representation of targeted C-, N-, P-, and S-cycling genes among the experimental configurations but do not directly indicate differences in gene expression or biogeochemical process rates.

#### 3.3.2 Diversity and composition of the targeted functional-gene panel

Changes in soil microbial functional gene diversity were assessed using alpha (α) and beta (β) diversity indices.

Alpha-diversity analysis (Figure 7A) showed lower Shannon and Sobs values in TO than in AT, by 1.23% and 4.97%, respectively (*P* < 0.05), whereas Pielou's evenness did not differ significantly (*P* > 0.05). Thus, the targeted functional-gene panel showed lower richness and diversity in TO at the 92-day endpoint.

**Figure 7:**
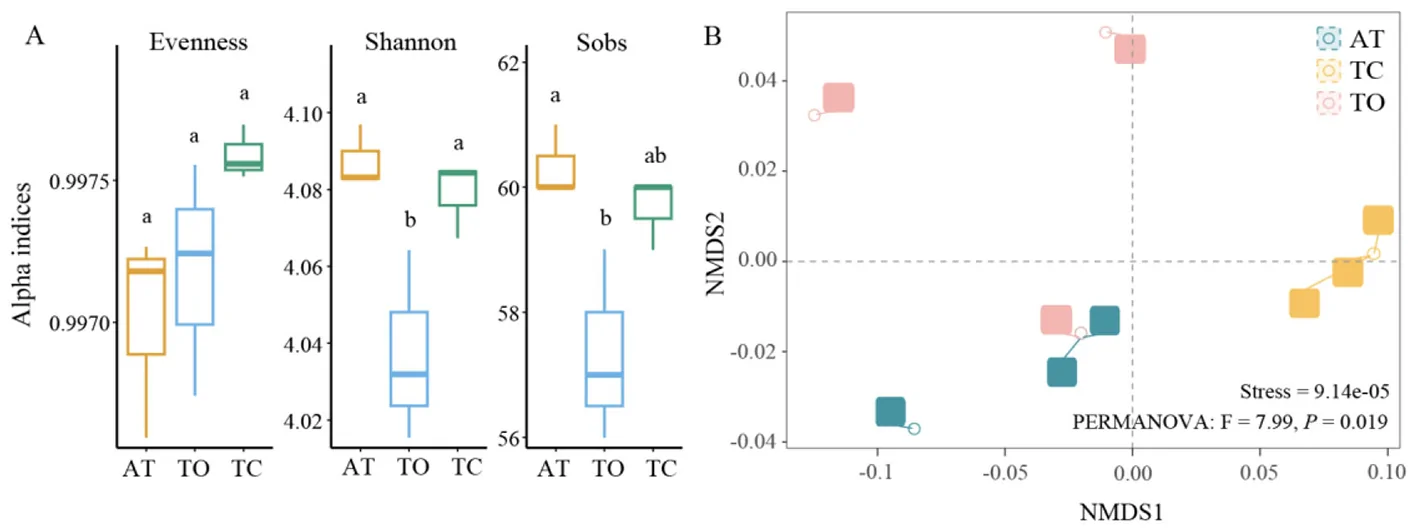
Variations in the diversity of soil microbial functional genes in the microbial fuel cell systems. **(A)** Alpha diversity of functional genes. **(B)** Beta diversity of functional gene communities. AT, non-electrode anaerobic control; TO, open-circuit conductive-material treatment; TC, closed-circuit treatment. Different lowercase letters indicate statistically significant differences among treatments (*P* < 0.05).

Beta diversity analysis (Figure 7B) further revealed treatment-dependent variations in functional gene community composition. Non-metric Multidimensional Scaling (NMDS) ordination showed that the TC was clearly separated from the other groups along the first axis. The functional gene communities clustered tightly within replicates but were clearly differentiated between treatments. This observation was statistically confirmed by PERMANOVA, which demonstrated a significant treatment effect (*P* < 0.05).

Overall, these findings indicate that the composition of the targeted functional-gene panel differed among the experimental configurations at the 92-day endpoint.

### 3.4 Variations in standardized targeted functional-gene summary scores

The overall standardized summary score of the targeted functional-gene panel did not differ significantly among treatments. The standardized P-cycling score was lower in TO than in AT, whereas the standardized S-cycling score was higher in TC than in the other treatments (Figure 8). Because these indices were derived from Z-score-transformed data and lack a natural zero point, percentage changes were not calculated.

**Figure 8:**
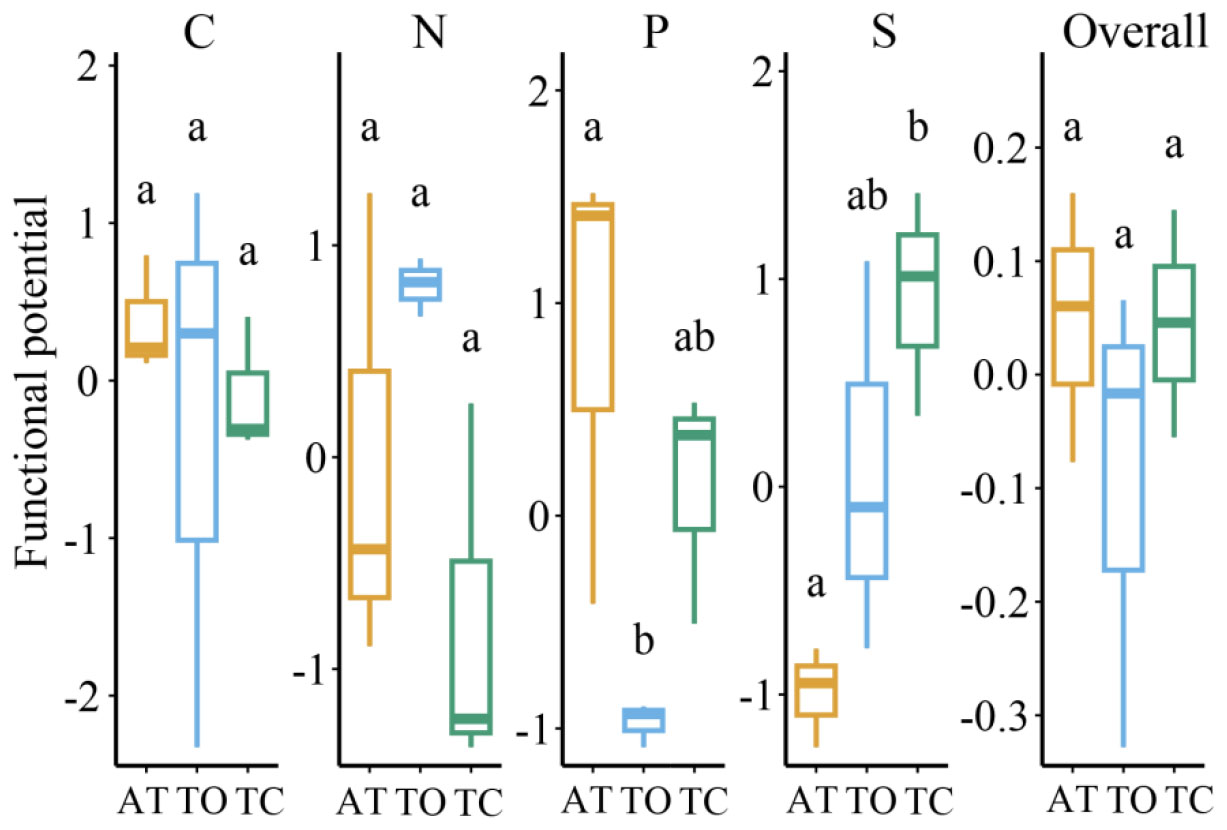
Standardized summary scores of the targeted functional-gene panel across the three experimental configurations. Scores were calculated as the mean Z-score-transformed relative abundances of measured genes associated with carbon (C), nitrogen (N), phosphorus (P), and sulfur (S) cycling. The overall score represents the mean standardized value across all detected target genes. These scores are descriptive summaries of the measured gene panel and should not be interpreted as direct measures of gene expression, process rates, or total microbial functional capacity. The horizontal line within each box indicates the median; box boundaries represent the 25th and 75th percentiles, and whiskers extend to 1.5 times the interquartile range. AT, non-electrode anaerobic control; TO, open-circuit conductive-material treatment; TC, closed-circuit treatment. Different lowercase letters indicate significant differences among treatments (*P* < 0.05).

## 4 Discussion

### 4.1 Closed-circuit operation was associated with changes in bacterial community assembly and targeted functional-gene profiles

Previous studies have already documented microbial community changes in soil bioelectrochemical systems under different circuit configurations. The contribution of the present study is therefore not the demonstration that open- and closed-circuit systems harbor different microbial communities, but the integration of null-model-based community assembly analysis with targeted functional-gene profiling across the AT, TO, and TC configurations (Zhao et al., 2019; Song et al., 2022). Within this framework, the AT–TO contrast describes responses associated with the combined addition of electrodes and short-cut carbon fibers, whereas the TO–TC contrast describes responses associated with circuit closure under otherwise identical conductive-material configurations. In the present study, the three experimental configurations provided a basis for distinguishing responses associated with conductive-material addition from those associated with circuit closure. The AT treatment contained neither electrodes nor short-cut carbon fibers, TO contained both conductive components under open-circuit conditions, and TC contained the same conductive-material configuration under closed-circuit conditions.

The comparison between TO and AT therefore reflects the combined introduction of electrodes and short-cut carbon fibers rather than the effect of electrode presence alone. Conductive materials can alter soil pore structure, attachment surfaces, microscale habitat heterogeneity, and potential pathways of interspecies electron transfer (Li et al., 2016; Chen et al., 2022). These physical and physicochemical changes may partly explain the differences in nutrient availability, targeted functional-gene diversity, and exploratory network topology observed between AT and TO. However, because the electrode materials and carbon fibers were added together, their individual contributions cannot be separated in the present design.

The comparison between TC and TO evaluates responses associated with circuit closure under otherwise identical conductive-material configurations. TC showed a higher estimated relative contribution of deterministic assembly processes, contained several characteristic taxa associated with electroactive metabolism, and showed higher relative abundances of several targeted functional genes. Closed-circuit bioelectrochemical systems can create distinct redox and energetic conditions that influence microbial colonization and community development (Zhang et al., 2020; Chang et al., 2022; Mahto and Das, 2025). The observed endpoint differences are therefore consistent with microbial responses to conditions associated with the closed-circuit configuration, although the temporal development of these differences was not resolved.

Nevertheless, voltage, current density, cumulative charge, electrode potential, and temporal variation in electrical output were not continuously quantified during the 92-day experiment. It is therefore not possible to relate the observed microbial responses to a measured electrical dose or to demonstrate that electric current alone caused these changes. The results should instead be interpreted as microbial responses associated with closed-circuit operation.

Characteristic taxa detected in TC included Geobacteraceae and Anaeromyxobacter. Members of these groups have previously been reported in electroactive biofilms and soil microbial fuel cells and may participate in extracellular electron transfer under suitable conditions (Zhao et al., 2019; Guo et al., 2024; Mahto and Das, 2025). Their occurrence in TC is consistent with the establishment of niches favorable to electroactive-associated taxa. However, taxonomic occurrence does not confirm active extracellular electron transfer. Direct evidence would require electrochemical characterization together with transcriptional, physiological, or isotope-based measurements.

### 4.2 Stochastic processes remained dominant despite a higher estimated deterministic contribution in TC

Microbial community assembly is generally governed by the combined effects of deterministic and stochastic processes, including environmental selection, ecological drift, dispersal limitation, homogenizing dispersal, and historical contingency (Stegen et al., 2013; Yuan et al., 2019; Zhou and Ning, 2017). In the present study, stochastic processes remained the dominant component of bacterial community assembly in AT, TO, and TC. The main treatment-related pattern was a higher estimated relative contribution of deterministic processes in TC than in TO. This result does not indicate replacement of stochastic assembly by deterministic assembly. Rather, at the 92-day endpoint, TC showed a higher estimated relative contribution of deterministic processes than TO, while stochastic processes remained dominant in all treatments.

The continued dominance of stochastic processes in all treatments is reasonable for soil microbial communities, which contain large regional species pools and are influenced by microscale dispersal, demographic variation, heterogeneous resource distributions, and priority effects (Zhou and Ning, 2017). The homogenization of the initial soil reduced some initial heterogeneity, but spatial variation could still develop within the reactors during the 92-day incubation. Such variation may have maintained a substantial stochastic component in community assembly.

The introduction of conductive materials in TO may have increased the number and heterogeneity of available microsites. Carbon cloth, activated-carbon cathode material, and short-cut carbon fibers can provide surfaces for attachment and modify microscale resource and electron-transfer conditions (Li et al., 2016; Chen et al., 2022). In the absence of a closed external circuit, these newly available microsites may be colonized through processes that retain a substantial stochastic component. This interpretation is broadly compatible with lottery-type community assembly, in which taxa capable of using similar niches may establish according to colonization history and local chance events (Verster and Borenstein, 2018). However, the current results do not directly test the lottery model.

The higher estimated deterministic contribution in TC may be related to environmental conditions associated with circuit closure. Closed-circuit operation can modify electron-acceptor availability, local redox gradients, and the energetic context of microbial metabolism (Zhang et al., 2020; Chang et al., 2022). These changes may favor taxa with traits compatible with electrode-associated or extracellular electron-transfer metabolism. The occurrence of Geobacteraceae and Anaeromyxobacter among the characteristic taxa of TC is consistent with this interpretation. Similar enrichment of electroactive-associated microorganisms has been reported in microbial fuel cells under different electrode configurations and applied potentials (Zhao et al., 2019; Guo et al., 2024; Chang et al., 2022).

This interpretation should remain cautious. The environmental-filtering concept describes a relationship between environmental conditions and the traits of organisms that persist, but community patterns alone cannot identify the exact selective mechanism (Kraft et al., 2015). The present study did not directly quantify redox potential, electrode potential, spatial oxygen gradients, extracellular electron-transfer rates, or the physiological traits of individual taxa. Circuit-associated environmental selection is therefore a plausible explanation for the null-model pattern rather than a directly demonstrated mechanism.

The β-diversity decomposition provided complementary information. The higher βNest value in TC than in TO indicates that a larger component of compositional dissimilarity was attributable to nestedness. This pattern suggests that some communities represented subsets of broader assemblages, potentially reflecting differential taxon retention or taxon loss. However, nestedness and deterministic assembly are distinct ecological properties. An increase in βNest cannot independently demonstrate stronger environmental filtering. In this study, the inference of a higher deterministic contribution is based primarily on the null-model analysis, whereas βNest describes the structure of compositional differences among samples.

### 4.3 Closed-circuit operation was associated with pathway-specific variation in targeted functional genes

The HT-qPCR analysis revealed treatment-dependent variation in the 16S-normalized relative abundances of the 71 targeted functional genes. In general, TC showed higher relative abundances of many measured carbon-, nitrogen-, and phosphorus-cycling genes than AT and/or TO. All detected sulfur-cycling genes also showed higher relative abundances in TC. These results indicate that the targeted DNA-level functional-gene profile differed among the experimental configurations.

The functional-gene results should be interpreted at the level of relative DNA abundance. The analysis did not measure RNA transcription, protein production, enzyme activity, substrate turnover, or elemental transformation rates. Consequently, terms such as “upregulation,” “gene expression,” and “metabolic activation” are not appropriate for these data. Higher 16S-normalized relative abundance indicates a greater representation of a target gene relative to the bacterial 16S rRNA gene but does not necessarily indicate that the corresponding pathway was more active.

The higher relative abundances of several carbon-cycling genes in TC suggest that closed-circuit conditions were associated with changes in the representation of genes related to carbon degradation, carbon fixation, and methane metabolism. Bioelectrochemical environments may influence carbon transformation by providing alternative electron-transfer routes and by changing the redox conditions experienced by microbial populations (Zhang et al., 2020; Chen et al., 2022). Nevertheless, the present study did not measure carbon mineralization, carbon fixation rates, methane production, or substrate-specific degradation. The functional consequences of these gene-abundance differences therefore remain uncertain.

A similar limitation applies to the nitrogen- and phosphorus-cycling genes. TC showed higher relative abundances of several genes involved in denitrification, nitrification, nitrogen fixation, organic nitrogen transformation, phosphorus solubilization, and organic phosphorus mineralization. The available soil measurements also showed treatment-related differences in alkali-hydrolyzable nitrogen and available phosphorus. However, changes in gene abundance cannot be directly linked to these nutrient concentrations because available nutrients are influenced by multiple biological and physicochemical processes. Sorption to carbon materials, redistribution within the reactor, changes in pH or microscale redox conditions, and microbial uptake could all contribute to the endpoint patterns.

The sulfur-cycling genes showed the most consistent treatment response. Genes associated with sulfur oxidation and sulfate reduction, including dsrA, dsrB, apsA, soxY, and yedZ, had higher relative abundances in TC. Sulfur transformation can be linked to electron transfer in anaerobic and bioelectrochemical systems, and some sulfur-associated microorganisms can use electrodes or conductive materials as part of their electron-transfer environment (Chen et al., 2022; Mahto and Das, 2025). The enrichment of electroactive-associated taxa in TC is consistent with a possible relationship between circuit closure and the representation of sulfur-related metabolic traits.

However, the present experiment did not measure sulfate, sulfide, elemental sulfur, sulfur oxidation, sulfate-reduction rates, or sulfur-gene transcription. The results therefore do not demonstrate that sulfur cycling was accelerated in TC. They show only that the relative representation of the measured sulfur-cycling genes was higher under the closed-circuit configuration. The potential coupling between electroactivity and sulfur metabolism should be treated as a hypothesis for subsequent process-based studies.

The standardized targeted functional-gene summary scores were used to describe coordinated variation within groups of measured genes. The absence of a significant treatment difference in the overall score suggests that the experimental configurations did not produce a uniform directional change across the entire panel. In contrast, category-specific differences indicate that particular groups of genes responded differently.

These summary scores have important limitations. Averaging Z-score-transformed values assumes equal contribution from each gene, although the measured genes differ in ecological role, abundance range, primer specificity, amplification efficiency, and relationship to process rates. The scores therefore represent descriptive indices of the targeted panel rather than direct measurements of total microbial functional capacity. Because Z scores lack a natural zero point, percentage increases or decreases cannot be meaningfully calculated from these values. Treatment differences should be reported using the standardized score values and statistical comparisons rather than percentage changes.

The present data also provide insufficient evidence for metabolic streamlining, functional specialization, or reduced functional redundancy. Functional specialization would require evidence that microbial resource allocation or expressed metabolic activity became concentrated in a narrower set of pathways. Functional redundancy would require evaluation of the relationship between taxonomic diversity and shared functional capacity across taxa (Louca et al., 2018). The current HT-qPCR panel does not resolve gene carriers, expression levels, or metabolic allocation. A more appropriate conclusion is that closed-circuit operation was associated with pathway-specific changes in the relative abundance of targeted functional genes.

### 4.4 Exploratory differences in bacterial association-network topology

The bacterial association networks differed descriptively among AT, TO, and TC. The TO network contained the highest number of retained nodes and edges and had the highest average degree. TC had fewer edges and a lower average degree than TO, whereas AT showed the highest modularity value. These patterns indicate that the inferred association structures were not identical among treatments.

Conductive materials may alter microbial spatial organization by providing additional colonization surfaces and changing microscale habitat structure (Li et al., 2016; Chen et al., 2022). The greater number of retained associations in TO may therefore be related to the combined presence of electrodes and carbon fibers. However, network edge number and average degree are affected by sample number, retained node number, abundance filtering, sparsity, and the statistical method used to infer associations. They cannot be interpreted as direct measurements of biological interaction strength.

Positive and negative network associations also require cautious interpretation. Positive associations may reflect similar environmental preferences, shared resource responses, common dispersal patterns, indirect dependencies, or compositional effects. Negative associations may reflect contrasting environmental responses, exclusion from shared habitats, or statistical constraints. These associations do not by themselves demonstrate cooperation or competition among microorganisms.

The current network analysis does not support conclusions that the TO network was fragile or that the TC network was more robust or stable. Ecological stability includes multiple properties, such as resistance, resilience, persistence, variability, and recovery after disturbance. None of these properties was directly measured in the present experiment. Although network modularity has been related to stability in some theoretical and empirical studies, the relationship depends on interaction strength, interaction type, network architecture, and environmental context (Grilli et al., 2016). A higher modularity value alone cannot establish greater ecological stability.

The modularity results also require accurate description. AT had the highest modularity, followed by TC and TO. Therefore, the results do not support a general conclusion that closed-circuit operation produced the most modular community. TC was more modular than TO, but less modular than AT. This pattern may indicate that conductive-material addition under open-circuit conditions was associated with a denser and less modular inferred network, whereas circuit closure was associated with an intermediate network structure. Because of the limited number of independent reactors and the nested spatial sampling design, these network differences should be regarded as exploratory.

Previous studies have shown that microbial network properties can vary across geographic gradients, environmental conditions, and microbial habitats (Ma et al., 2016). However, statistically stable treatment-specific networks generally require substantially more independent samples than were available here. The present networks are therefore best used to visualize possible association patterns and to generate hypotheses for future experiments rather than to establish treatment effects on microbial interactions or stability.

### 4.5 Potential implications for soil biogeochemical cycling and bioelectrochemical remediation

The combined taxonomic, assembly, functional-gene, and physicochemical results indicate that conductive-material addition and closed-circuit operation were associated with different aspects of the soil microbial system. TO differed from AT in targeted functional-gene diversity, nutrient availability, and exploratory network topology. TC differed from TO in the estimated relative contribution of deterministic assembly, the occurrence of several electroactive-associated taxa, and the relative abundance of multiple targeted functional genes. A conceptual summary of the observed microbial community and targeted functional-gene patterns across the three experimental configurations is presented in Figure 9.

**Figure 9:**
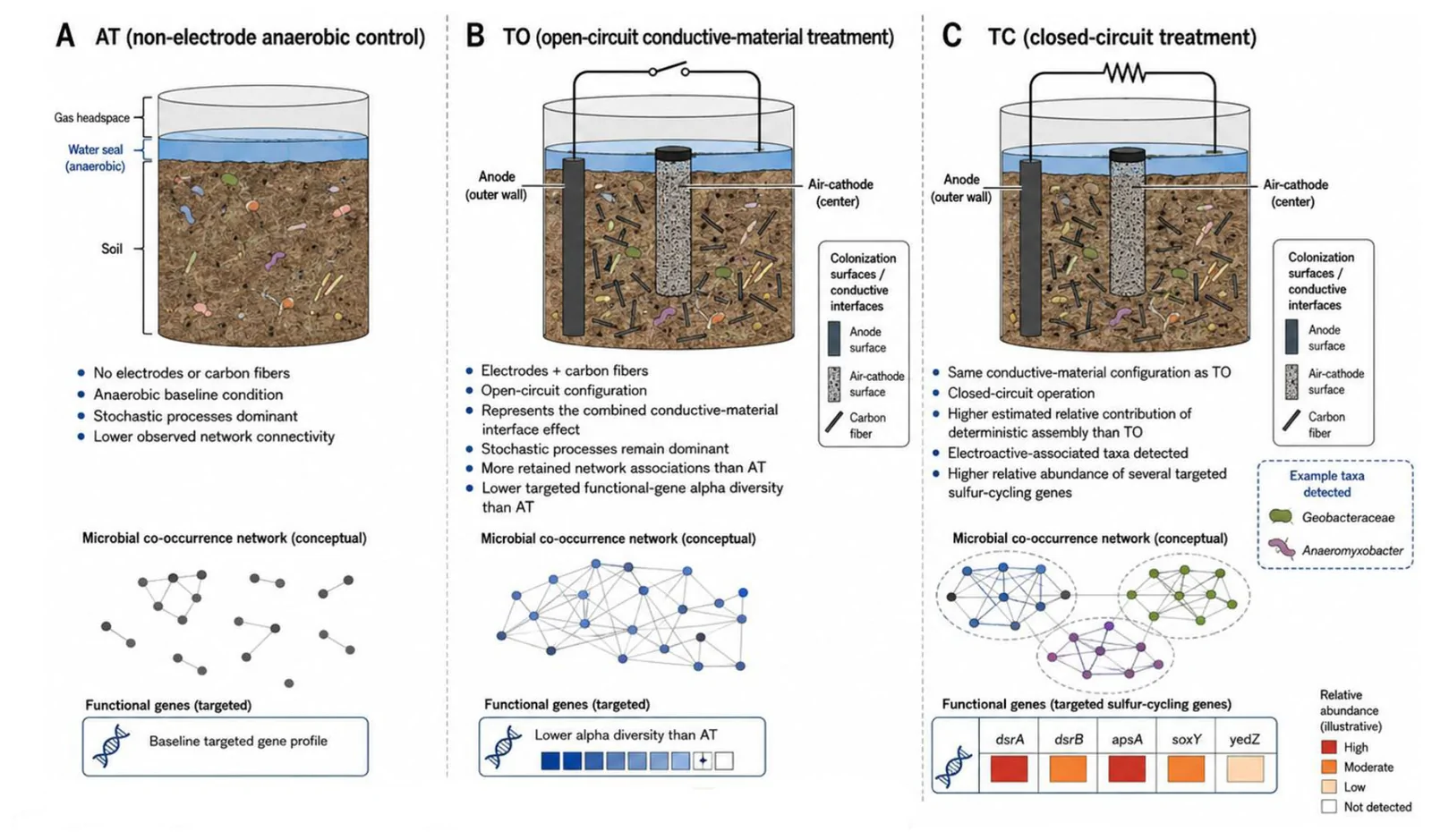
**(A)** AT, non-electrode anaerobic control; **(B)** TO, open-circuit conductive-material treatment; and **(C)** TC, closed-circuit treatment.

The differences between TO and AT suggest that adding conductive materials may influence the soil environment even without a closed external circuit. Carbon fibers and electrode materials can alter attachment surfaces, microscale habitat heterogeneity, conductivity, and potential pathways of direct or indirect interspecies electron transfer (Li et al., 2016; Chen et al., 2022). These changes may influence microbial distribution and nutrient availability. Nevertheless, the design cannot separate the effect of the electrode surfaces from that of short-cut carbon fibers.

The differences between TC and TO suggest that circuit closure introduced an additional environmental factor beyond conductive-material addition. Previous studies have reported that applied potentials and closed-circuit operation can influence the temporal development and composition of electroactive biofilms (Chang et al., 2022; Guo et al., 2024). However, because the present study included only a single endpoint, it cannot determine when these treatment-associated differences emerged or how they developed during the incubation period. In the present experiment, the higher estimated deterministic contribution and the occurrence of electroactive-associated taxa in TC are consistent with this general pattern. The higher relative abundances of several sulfur-cycling genes also provide a hypothesis that sulfur-associated traits may be favored under closed-circuit conditions.

These microbial patterns may be relevant to the use of soil microbial fuel cells for remediation. Previous studies have shown that soil bioelectrochemical systems can remove antibiotics and antibiotic resistance genes under some operating conditions (Zhao et al., 2019; Song et al., 2022), while microbial fuel cells have also been investigated for the treatment of antibiotic-containing wastewater (Li et al., 2022). However, the present study did not measure tetracycline removal, degradation kinetics, transformation products, residual toxicity, or antibiotic resistance genes. It therefore cannot determine whether the observed microbial changes improved tetracycline remediation.

The enrichment of taxa associated with electroactive metabolism also cannot be assumed to explain contaminant degradation. Electroactive organisms may participate in electron transfer, but their functional role depends on electrode potential, available substrates, redox conditions, community interactions, and contaminant chemistry (Mahto and Das, 2025). Directly linking community composition to remediation performance would require simultaneous measurements of electrical output, pollutant transformation, metabolic activity, and microbial gene expression.

The endpoint differences in available phosphorus and alkali-hydrolyzable nitrogen similarly cannot be assigned to specific microbial pathways. Nutrient availability may be affected by microbial mineralization, immobilization, redox-dependent transformation, sorption to activated carbon or carbon fibers, and spatial transport within the reactor. Further experiments should include measurements of dissolved and total nutrient pools, relevant intermediate compounds, process rates, and spatial chemical gradients.

### 4.6 Limitations

This study has several limitations. First, the reactor was the experimental unit, whereas samples collected at the cathode surface, midpoint, and anode surface represented within-reactor spatial subsamples. The limited number of independently operated reactors restricts the strength of treatment-level statistical inference, and the results should therefore be interpreted as exploratory. Second, the TO–AT comparison reflects the combined introduction of electrodes and short-cut carbon fibers; the individual effects of these conductive materials cannot be separated. Third, continuous cell voltage, current density, cumulative charge, individual electrode potentials, and temporal electrical-output profiles were not quantified throughout the 92-day operation. Consequently, the TC–TO comparison identifies responses associated with closed-circuit configuration but does not allow the observed microbial changes to be related to a quantified electrical dose, electrode potential, or temporal pattern of current generation. Future studies should incorporate continuous voltage monitoring, reference-electrode measurements, current-density and cumulative-charge calculations, and reactor-level electrochemical replication. Fourth, the targeted qPCR panel describes DNA-level relative gene abundance and does not directly measure transcription, enzyme activity, metabolic flux, or biogeochemical process rates. Fifth, tetracycline removal, transformation products, and toxicity were not measured, preventing a direct link between microbial changes and remediation performance. Finally, sampling at a single endpoint precludes inference regarding temporal succession. Future studies should use additional independent reactors, factorial controls separating electrodes from carbon fibers, continuous electrochemical monitoring, time-series sampling, and direct measurements of pollutant transformation and process rates.

## Data Availability

The original contributions presented in the study are included in the article/Supplementary material, further inquiries can be directed to the corresponding author.
