# Supplementary material for: Closed-circuit bioelectrochemical operation is associated with a higher relative contribution of deterministic assembly and altered targeted functional-gene profiles in soil microbiomes
Source: Front Microbiol. 2026 Oct 9;17:1815770. doi: 10.3389/fmicb.2026.1815770 (PMC13690965; doi:10.3389/fmicb.2026.1815770)
Supplement: Supplementary file 1 [file Data_Sheet_1.pdf]

Table S1 List of primers for functional genes

Gene name	Function classification	Forward sequence	Reverse sequence
amyA	C degradation	YGGTTTTCGTCTTGACGCSG	MGGCTGMGTRTCATGRTTK
amyX	C degradation	TATAAYTGGGGMTATGAYCC	CCCATYAAATCAAAWCGRAA
apu	C degradation	ACVTGGATAGGYGAGCCYCA	CCRTCSGGGAAGTAGTTKCC
iso-plu	C degradation	GTCATYTACTTYGGNCC	CGNGCSACATCNGCCCA
sga	C degradation	CGSAACTGGGAYTACCGS	TCCCACAGSCCSKCGTC
pgu	C degradation	ANCATTGGTGGCCSTGGAA	TTRAYGGCRATRCARTCRTC
glx	C degradation	AACCAGTCGATCATCTACGA	RTGSACGAGCTCDGGCATGG
lig	C degradation	CCGCACACACTGTTGCTGC	CGAAGGATTGCCACTCGCA
mnp	C degradation	MACRCCSTTCGACTCSACC	ACGTCSGAGCAGTCRAYGA
pox	C degradation	ACYAGTATCCATTGGCACGGT	AGATGVGARTGATACCARAA
abfA	C degradation	CGSTAYCCSGGCGGCAAYTT	TGCCASGGNCCGTCCATYTC
manB	C degradation	ATGCGCGGBGTCAACCA	TCGTTGSCGATGTTGABGA
xylA	C degradation	TGGGGBGGTCGYGAAGG	ACTTTGGCRTCRAAGTT
chiA	C degradation	TSAAGAARTACGCSGACAACG	ASGTCATCAGRCCCTTSAG
exo-chi	C degradation	GATTGGTSVCAATATGAYRG	STCCARCCACCRAYRCTRAA
cdh	C degradation	ATWRYCTWCCGMRTHGCCMT	GTKAGSGGRTTBYKGRYCAT
cex	C degradation	YSTACGGSATGCACTGGMT	TANCGCAGRTAGTCVCCCAT
naglu	C degradation	TVAAYTGGTAYCTGAAATAY	CCRTGYAGVGCCATCCAGTC
accA	C fixation	GAAGGCTAYCGCAARGC	CCTTCMGGSGARATMAC
aclB	C fixation	TGGACMATGGTDGCYGGKGGT	ATAGTTKGGSCCACCTCTTC
acsA	C fixation	GATACCTGGTGGCAGACCGA	TGATCACGTCGTCGACCCGG
acsB	C fixation	CTYTGYCAGTCMTTYGCBCC	CCCATAAABCCYGGDGTYTG
acsE	C fixation	TCATCGGCGAACGCATCAAC	AGRCCGGCTTCSATGGC

Gene name	Function classification	Forward sequence	Reverse sequence
cdaR	C fixation	CGARATGGTGGTGCTCAA	CARCGTRTTACGATGAATA
frdA	C fixation	MTGCTGCACACSCTGTW	CCGGTSGGGTGRWACTG
korA	C fixation	GCCGGCTACCCCATCACCCC	ATGATGGGATGGTCGCCATG
mct	C fixation	TGGGCGCSGASGTSATMCG	TTGACSGTRTARTCSAYSGC
pccA	C fixation	GTGMTGATCAAGGCCWC	CGSGTGTTCATYTCSAGGAA
rbc L	C fixation	AAGGAYGACGAGAACATC	TGCAGSATCATGTCRTT
smtA	C fixation	TTTCTGGCCGGBTAYGCDGC	CGGTACGGHCCGGTYTGVCC
mcrA	Methane metabolism	GGTGGTGTMGGDTTCACMCARTA	CGTTCATBGCGTAGTTVGGRTAGT
mmoX	Methane metabolism	ATGGAGGCGGTCAAGGACGA	CGCTTCATGCCCTTCCACAG
mxaF	Methane metabolism	GCGGCACCAACTGGGGCTGGT	GGGCAGCATGAAGGGCTCCC
pqq-mdh	Methane metabolism	TGTTCTATGTGCCGGCCAA	CTTCCACAGTTCCTTGCC
pmoA	Methane metabolism	GGNGACTGGGACTTCTGG	GAASGCNGAGAAGAASGC
amoA1	N Cycling	STAATGGTCTGGCTTAGACG	GCGGCCATCCATCTGTATGT
amoA2	N Cycling	GGGGTTTCTACTGGTGGT	CCCCTCKGSAAAGCCTTCTT
amoB	N Cycling	TGGTAYGACATKAWATGG	RCGSGGCARGAACATSGG
gdhA	N Cycling	GCCATCGGYCCWTACAAGGG	ATGTCRCCNGCCGGAACGTC
hao	N Cycling	TGTCACATGGGTGTAGACCA	ACCTGGAACATACCCAT
hzo	N Cycling	AAGACNTGYCAYTGGGGWAAA	GACATACCCATACTKGTRTANACNGT
hzsA	N Cycling	WTYGGKTATCARTATGTAG	AAABGGYGAATCATARTGGC
hzsB	N Cycling	ARGGHTGGGGHAGYTGGAAG	GTYCCHACRTCATGVGTCTG
napA	N Cycling	CTGGACIATGGGYTTIAACCA	CCTTCYTTYTCIACCCACAT
narG	N Cycling	TAYGTSGGGCAGGARAAACTG	CGTAGAAGAAGCTGGTGCTGT
nasA	N Cycling	CARCCNAAYGCNATGGG	ATNGTRTGCCAYTGRTC
nifH	N Cycling	AAAGGYGGWATCGGYAARTCCACCAC	TGSGCYTTGTCYTCRCGGATBGGCAT
nirK1	N Cycling	GGMATGGTKCCSTGGCA	GCCTCGATCAGRTTRTGGTT

Gene name	Function classification	Forward sequence	Reverse sequence
nirK2	N Cycling	ATGGCGCCATCATGGTNYTNCC	TCGAAGGCCTCGATNARRTTRTG
nirK3	N Cycling	TGCACATCGCCAACGGNATGTWYGG	GGCGCGGAAGATGSHRTGRTCNAC
nirS1	N Cycling	GTSAACGTSAAGGARACSGG	GASTTCGGRTGSGTCTTGA
nirS2	N Cycling	ATCGTCAACGTCAARGARACVGG	TTCGGGTGCGTCTTSABGAASAG
nirS3	N Cycling	TGGAGAACGCCGGNCARGTNTGG	GATGATGTCCACGGCNACRTANGG
nosZ1	N Cycling	CGCRACGGCAASAAGGTSMSSGT	CAKRTGCAKSGCRTGGCAGAA
nosZ2	N Cycling	CGYTGTTCMTCGACAGCCAG	CGSACCTTSTTGCCSTYGCG
nxrA	N Cycling	CAGACCGACGTGTGCGAAAG	TCCACAAGGAACGGAAGGTC
ureC	N Cycling	AAGMTSCACGAGGACTGGGG	AGRTGGTGGCASACCATSAGCAT
bpp	P Cycling	GACGCAGCCGAYGAYCCNGCNITNTGG	CAGGSCGCANRTCIACRTTRTT
cphy	P Cycling	GTGGACCTRCGRMARGARWCICA	GTCCGACCATTGCCTGCYTCRCARTGRAMRTGIADCCA
gcd	P Cycling	ATCGCGTTCGGGCCGGACG	ATSAGRTTSAGCTCGTCCCA
phnK	P Cycling	CATCGTCGGCGAATCCGG	TGCTGCATGCCGCCGGAAAA
phoD	P Cycling	CAGTGGGACGACCACGAGGT	GAGGCCGATCGGCATGTCG
phoX	P Cycling	GARGAGAACWTCCACGGYTA	GATCTCGATGATRTGRCCRAAG
ppk	P Cycling	GACCCGAABGTRCTBGCSAT	TTATAATTNCCSGTNCCNA
ppx	P Cycling	TGCATCTGGCGGACGGCCT	AGATCCGCCGCCAATATCA
pqqC	P Cycling	AACCGCTTCTACTACCAG	GCGAACAGCTCGGTCAG
apsA	S Cycling	GGGYCTKTCCGCYATCAAYAC	ATCATGATCTGCCAGCGGCCGGA
dsrA	S Cycling	ACSCACTGGAAGCACG	GGTGGAGCCGTGCATGTT
dsrB	S Cycling	CAACATCGTYCAYACCCAGGG	GTGTAGCAGTTACCGCA
soxY	S Cycling	ATCGATGACAACCCCGTGCC	AGCTGGTCCATCTGCATGCCG
yedZ	S Cycling	CTGCTGATCACGCTGGCCAT	GCGATGCAGCTTCTTCCAGCG

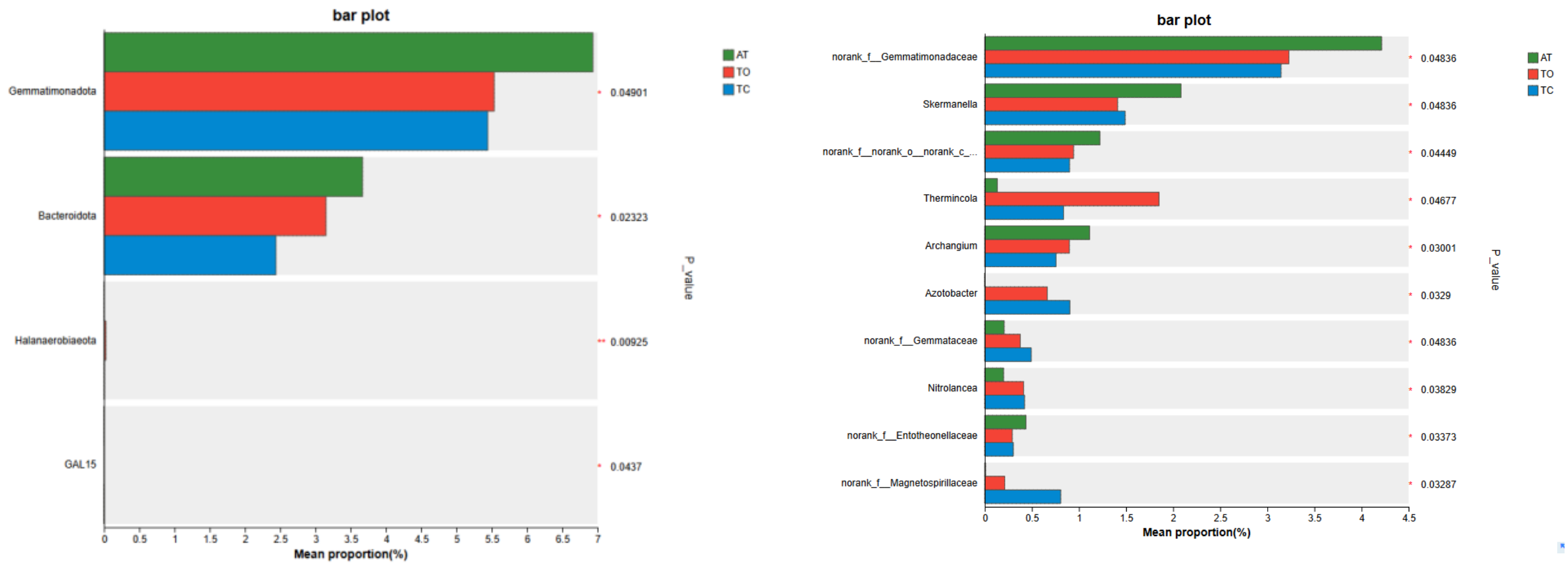

Figure S1. Changes in bacterial communities across different treatments in the microbial fuel cell system.

(A) Variations in the relative abundance of bacterial communities at the phylum level among treatments. (B) Variations in the relative abundance of bacterial communities at the genus level among treatments. Abbreviations: AT, non-electrode control; TO, open-circuit control; TC, closed-circuit treatment.

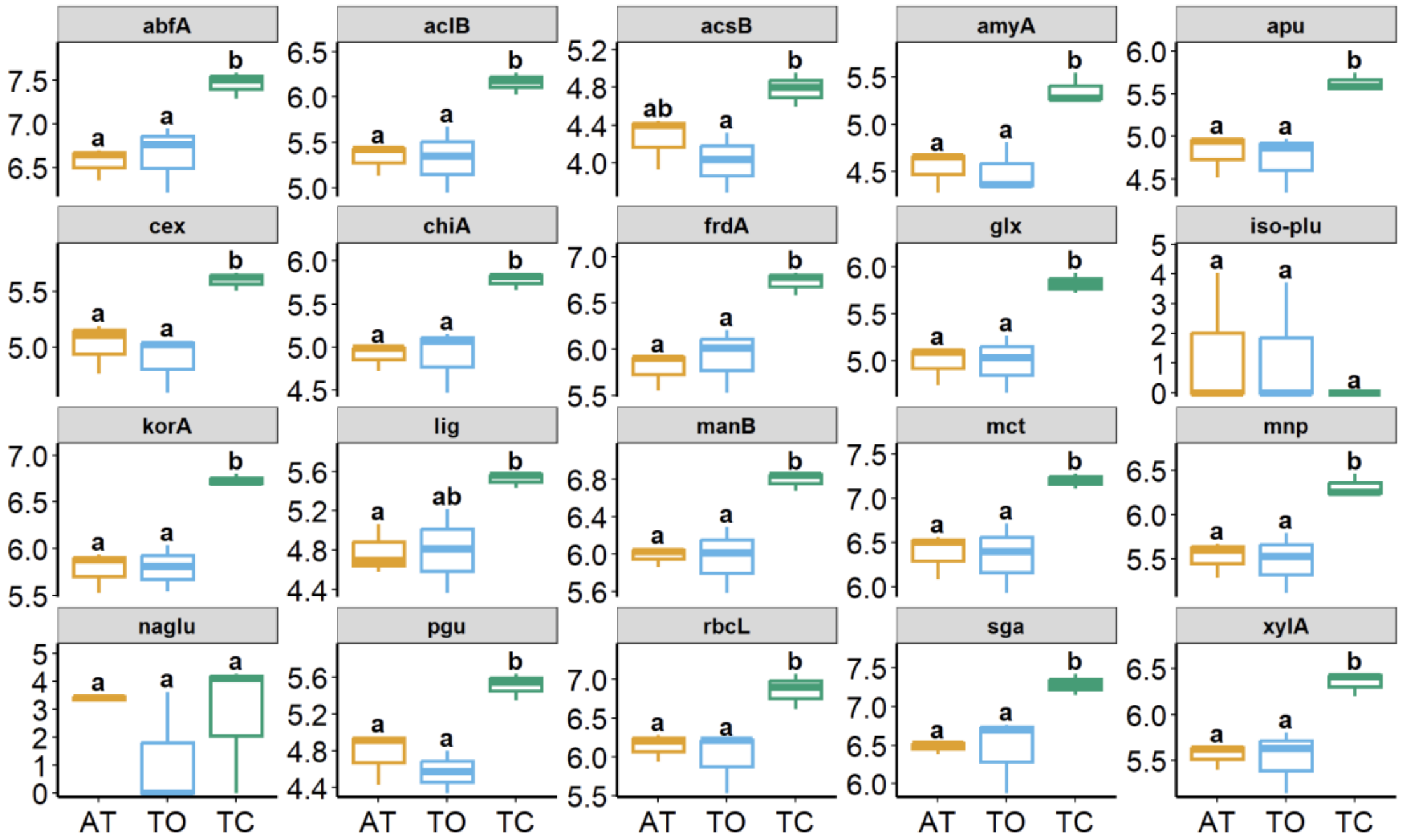

Figure S2 Relative contributions of stochastic and deterministic processes to bacterial community assembly.

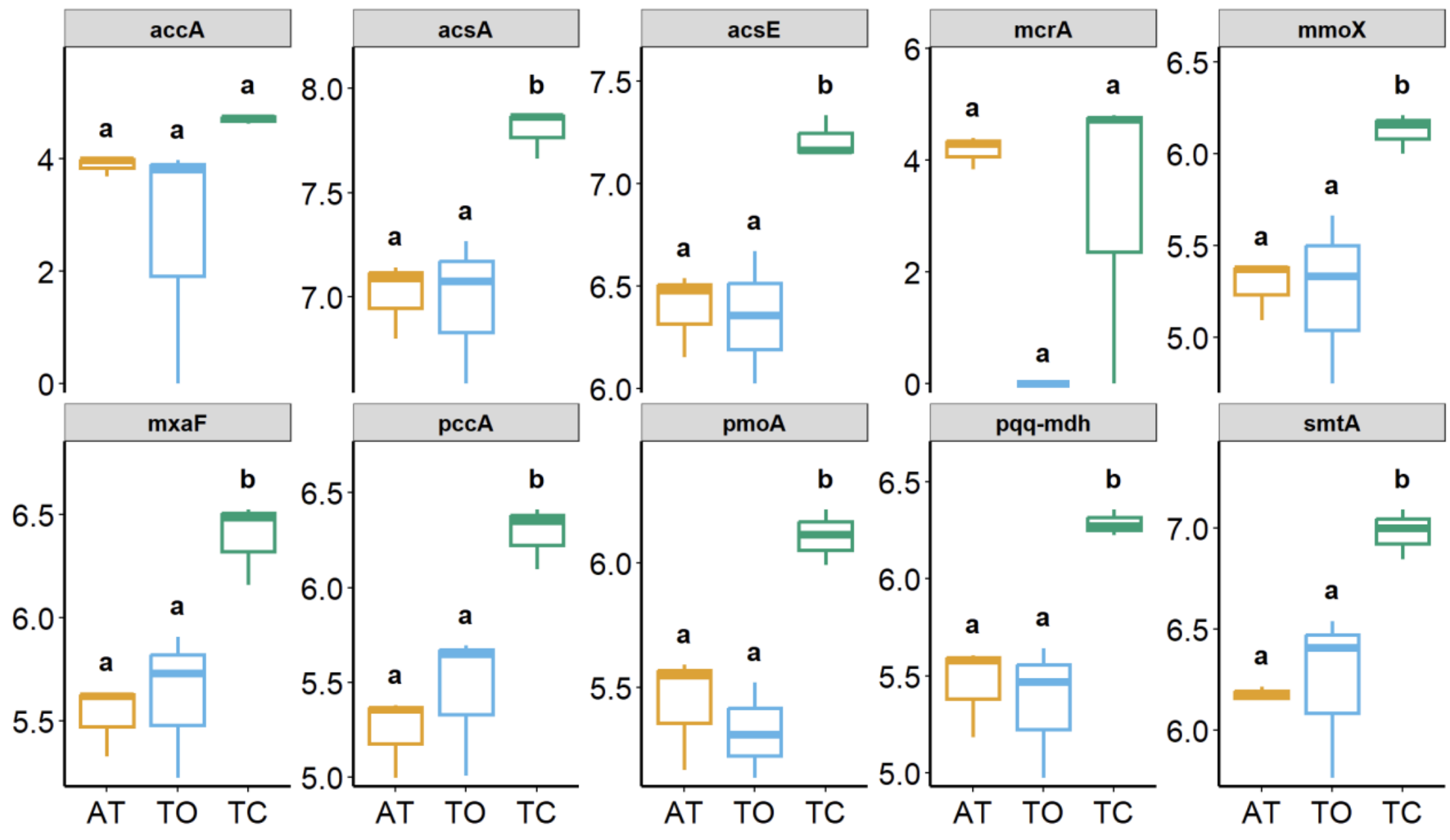

Figure S3 Variations in carbon-cycling functional genes in the microbial fuel cell system.

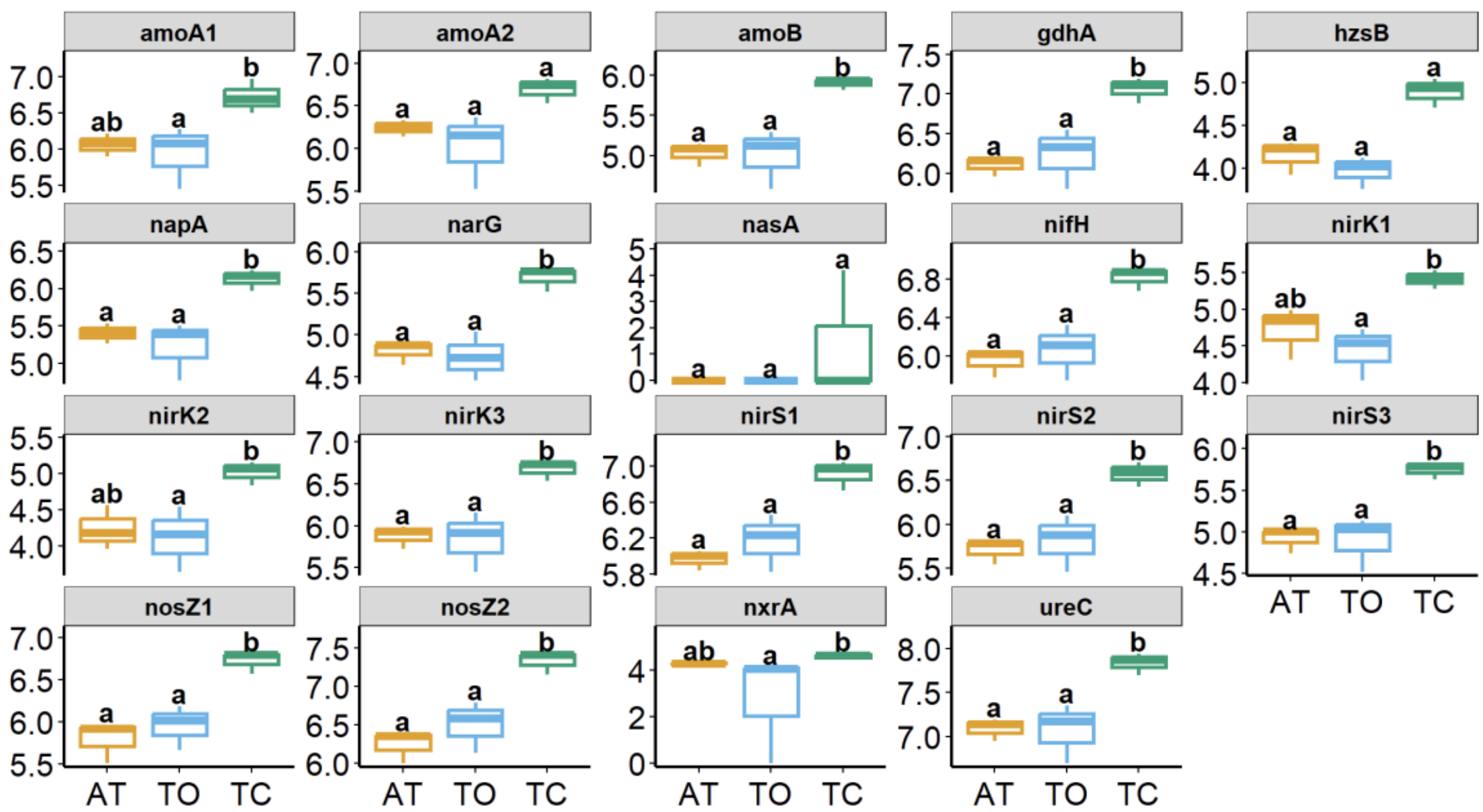

Figure S4 Variations in nitrogen-cycling functional genes in the microbial fuel cell system.

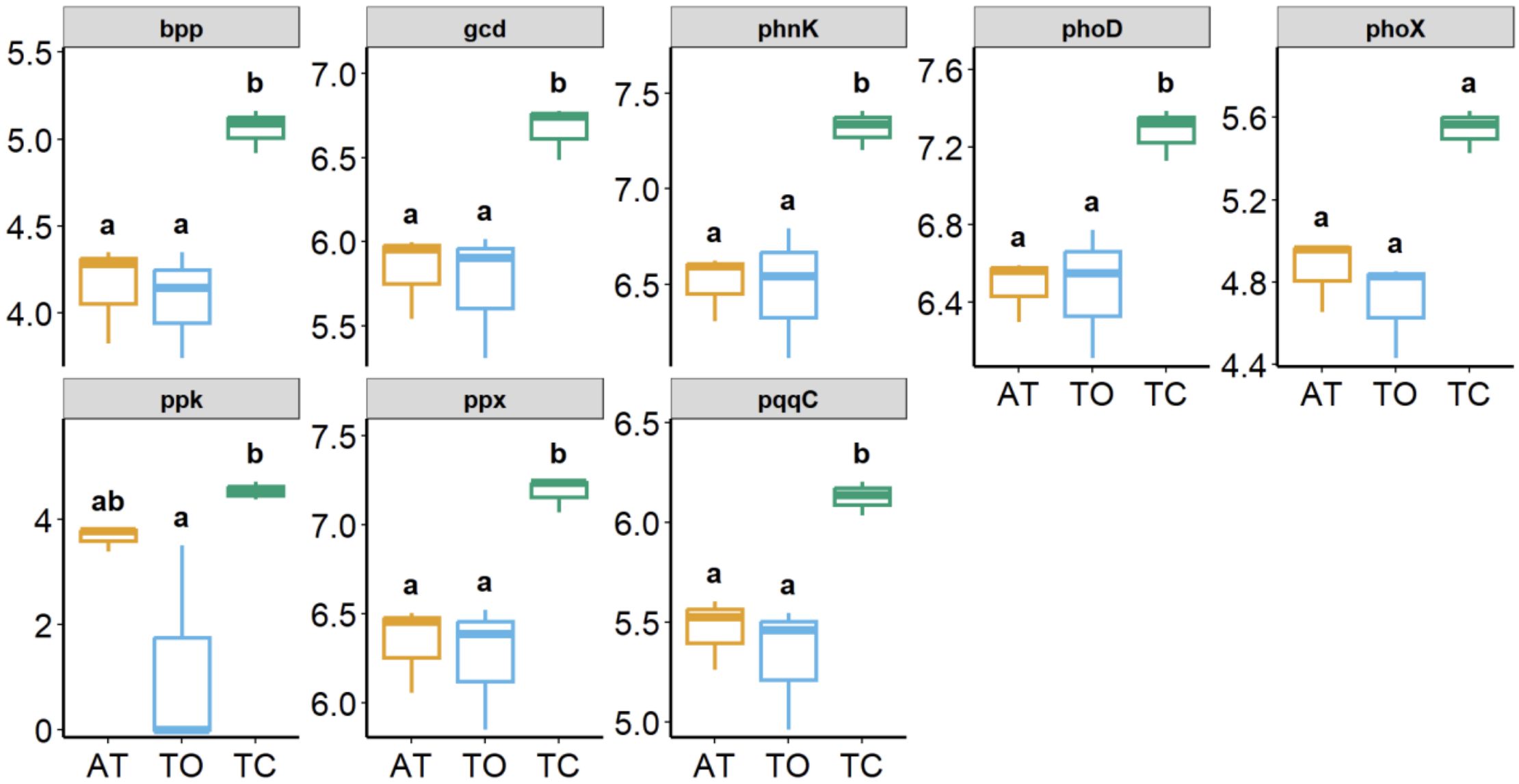

Figure S5 Variations in phosphorus-cycling functional genes in the microbial fuel cell system.

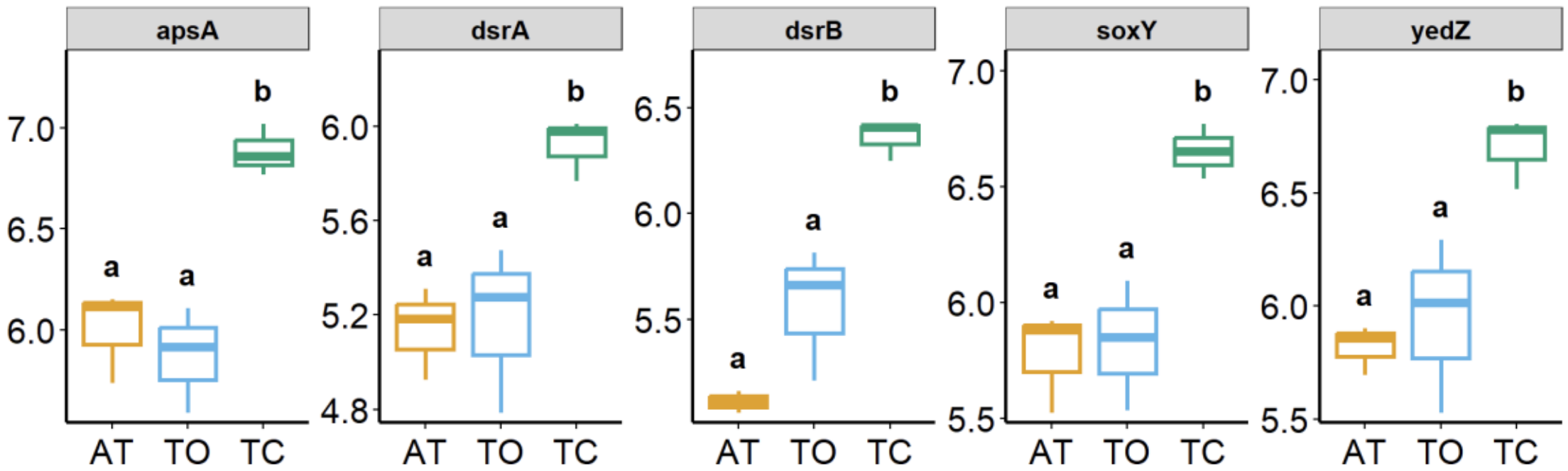

Figure S6 Variations in sulfur-cycling functional genes in the microbial fuel cell system.
